# Using Co-design in Mobile Health System Development: A Qualitative Study With Experts in Co-design and Mobile Health System Development

**DOI:** 10.2196/27896

**Published:** 2021-11-10

**Authors:** Tyler J Noorbergen, Marc T P Adam, Timm Teubner, Clare E Collins

**Affiliations:** 1 School of Information and Physical Sciences College of Engineering, Science and Environment University of Newcastle Callaghan Australia; 2 Technical University Berlin Berlin Germany; 3 Hunter Medical Research Institute New Lambton Australia; 4 School of Health Sciences College of Health, Medicine, and Wellbeing University of Newcastle Callaghan Australia

**Keywords:** co-design, mHealth, guidelines, qualitative study, mobile phone

## Abstract

**Background:**

The proliferation of mobile devices has enabled new ways of delivering health services through mobile health systems. Researchers and practitioners emphasize that the design of such systems is a complex endeavor with various pitfalls, including limited stakeholder involvement in design processes and the lack of integration into existing system landscapes. Co-design is an approach used to address these pitfalls. By recognizing users as experts of their own experience, co-design directly involves users in the design process and provides them an active role in knowledge development, idea generation, and concept development.

**Objective:**

Despite the existence of a rich body of literature on co-design methodologies, limited research exists to guide the co-design of mobile health (mHealth) systems. This study aims to contextualize an existing co-design framework for mHealth applications and construct guidelines to address common challenges of co-designing mHealth systems.

**Methods:**

Tapping into the knowledge and experience of experts in co-design and mHealth systems development, we conducted an exploratory qualitative study consisting of 16 semistructured interviews. Thereby, a constructivist ontological position was adopted while acknowledging the socially constructed nature of reality in mHealth system development. Purposive sampling across web-based platforms (eg, Google Scholar and ResearchGate) and publications by authors with co-design experience in mHealth were used to recruit co-design method experts (n=8) and mHealth system developers (n=8). Data were analyzed using thematic analysis along with our objectives of contextualizing the co-design framework and constructing guidelines for applying co-design to mHealth systems development.

**Results:**

The contextualized framework captures important considerations of the mHealth context, including dedicated prototyping and implementation phases, and an emphasis on immersion in real-world contexts. In addition, 7 guidelines were constructed that directly pertain to mHealth: understanding stakeholder vulnerabilities and diversity, health behavior change, co-design facilitators, immersion in the mHealth ecosystem, postdesign advocates, health-specific evaluation criteria, and usage data and contextual research to understand impact.

**Conclusions:**

System designers encounter unique challenges when engaging in mHealth systems development. The contextualized co-design framework and constructed guidelines have the potential to serve as a shared frame of reference to guide the co-design of mHealth systems and facilitate interdisciplinary collaboration at the nexus of information technology and health research.

## Introduction

### Background

The proliferation of mobile devices (eg, smartphones and tablets) has enabled new ways of delivering health services via mobile health (*mHealth*) systems [1,2]. Broadly, mHealth can be defined as “medical and public health practice supported by mobile devices, such as mobile phones, patient monitoring devices, personal digital assistants (PDAs), and other wireless devices” [3]. The ubiquity and increasing capabilities of these systems have created enormous potential to support individuals in self-managing existing health conditions (eg, diabetes and stroke) and reducing their health risks by supporting healthier lifestyle habits (eg, increasing vegetable intake). The adoption of mHealth systems is steadily growing. In 2018, nearly half of the consumers in health care used mHealth systems compared with one-sixth in 2014. Overall, the global mHealth market is expected to grow from US $28.320 billion in 2018 to US $102.35 billion by 2023 [4].

Researchers have repeatedly emphasized that mHealth systems design and development is a complex endeavor with a range of pitfalls limiting adoption and/or effective usage in practice [5]. This is because the design process commonly entails limited stakeholder involvement [5,6], and solution artifacts lack integration with other health systems or their components [7]. To address these complexities, scholars have suggested co-design for mHealth systems development. Co-design refers to “the creativity of designers and people not trained in design, working together in the design development process” [8]. Research has referred to two main reasons for using co-design: (1) mHealth is a complex environment that requires the involvement of diverse stakeholders (eg, consumers/end users, government, health practitioners, scientists, and software developers) with co-design facilitating necessary collaborations [1,9,10]; (2) using co-design ensures that mHealth systems are underpinned by expert insights and best practices [5,11,12].

Despite repeated calls to use co-design for mHealth systems development [5,6], there is only limited guidance available on how to do so. The existing literature on co-design methodology provides important *general* guidance for the application of co-design frameworks and methods [8,13,14]. However, given the complexities surrounding a person’s health and the multitude of stakeholders, there is a need for research that identifies the *specific* challenges system designers face when applying co-design in mHealth and to illustrate ways in which these challenges can be addressed. As such, there is a lack of guidance in the current literature in terms of how one can apply co-design in the mHealth systems context.

### Objective

In this paper, we address this research gap by conducting a qualitative study that explores how co-design can be used in mHealth systems development. Specifically, we conducted 16 semistructured interviews to synthesize the theoretical and practical expertise of 8 co-design method experts (CMEs) and 8 mHealth system developers (MSDs) in a rapidly growing application area. Interviews were transcribed and analyzed using thematic analysis [15]. Thereby, the overarching research objectives of this study were (1) to contextualize an existing co-design framework for mHealth applications and (2) to construct guidelines to address common challenges of using co-design in mHealth development.

### Theoretical Background and Related Work

#### Related Work on mHealth Systems Design

mHealth systems have become a growing area for research and practice [16,17]. The two primary application domains that have emerged are (1) disease management and (2) health promotion. First, disease management empowers patients to manage their medical conditions more effectively and independently (eg, controlling blood sugar levels [18,19]). Second, health promotion facilitates better health choices by providing support and encouragement for users to engage in behaviors to lower risk factors and improve health (eg, better diet and smoking cessation). The design of mHealth systems is complex, with a range of pitfalls including limited stakeholder involvement [5], lack of integration with other health systems [7], and disregard of behavior change techniques [5].

Several studies have sought to improve mHealth systems design. McCurdie et al [20] discussed a user-centered design approach for mHealth systems development. The term user-centered design refers to “a design philosophy that places the needs, wants, and limitations of end users at the center of the design process” [21]. However, it should be noted that user-centered design adopts an expert perspective where “trained researchers observe and/or interview largely passive users” [8]. In contrast, in co-design, the user is in the position of being an expert of their own experience and actively plays a “large role in knowledge development, idea generation, and concept development” [8]. Banos et al [22] developed an architecture that showed how specific functionalities and components of mHealth systems could be implemented. Building on a user-centered design, Schnall et al [23] developed a 3-cycle framework (relevance, design, and rigor) to better incorporate end users’ preferences. Eckman et al [1] developed an mHealth systems framework that considers design thinking principles, using “a hypothesis-driven method of generating and validating new concepts” [1]. Nahum-Shani et al [24] explored the design of just-in-time adaptive interventions to support users’ health behavior change. However, there has been limited focus on how to apply a co-design approach that involves stakeholders within the mHealth context.

#### Co-design Frameworks

**Figure 1:**
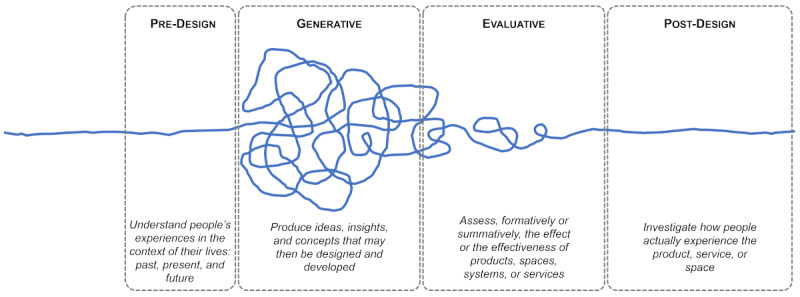
Co-design framework.

Researchers have proposed several frameworks to facilitate co-design [8,13,14]. By creating a conceptual structure of the process, these frameworks provide a shared frame of reference for researchers and practitioners engaging in system design. As noted by Sanders and Stappers [13], these frameworks can be understood as a response to the increased attention to methods: “So many methods, tools and techniques have been introduced that it has become useful to provide frameworks for organizing them” [13]. For instance, the framework by Visser et al [14] structured the co-design process into five phases: preparation, sensitization, sessions, analysis, and communication. Brandt et al [25] described an iterative cycle of making, telling, and enacting. Building on these earlier conceptualizations, the framework by Sanders and Stappers [13] has become one of the most widely recognized co-design resources (538 citations on Google Scholar, February 2021). The framework breaks down the timeline of the co-design process (shown in blue) into 4 interconnected phases (Figure 1, adapted from a study by Sanders and Stappers [13]).

1. The predesign phase is concerned with understanding the surrounding context and people’s experiences, exploring knowledge in the user context, establishing goals for future experiences, and sensitizing participants to the problem space [8,13].
2. The generative phase focuses on producing ideas, insights, and concepts that explore the *design space*, with users taking an active role in making through co-creation of conceptual artifacts (eg, journey maps, mock-ups, and storyboards). Although the vision is still fuzzy, these activities test, transform, and refine *ideas, insights, and concepts that may then be designed and developed* [13].
3. The evaluative phase allows users to assess the effects and effectiveness of the devised concepts. The vision of the final artifact becomes more tangible through the evaluation prototypes that allow users *to experience a situation that did not exist before* [13].
4. The postdesign phase captures the notion that once a system is part of a user’s lived experiences, it needs to evolve along with their needs, habits, and use patterns. Hence, *the tail end of the postdesign phase [leads] to the front end of another design process* [13].

#### Co-design in mHealth

In recent years, an increasing number of mHealth studies have used a co-design approach. A 2016 review [26] identified early mHealth studies that used co-design with many following an approach similar to the framework by Sanders and Stappers [13]. A 2021 review [27] documented the application contexts (eg, diabetes and nutrition), stakeholders (eg, caregivers, nurses, and specialists), and methods used in co-design mHealth studies, including a mapping to the Sanders and Stappers framework [13]. The methods that have been applied include cultural probes [28-31], storytelling [32-36], and journey maps [31,37,38]. The context of these applications includes both disease management and health promotion. Examples from the disease management context include diabetes [30,39], cancer [40], asthma [41,42], heart failure [43-45], and depression [46]. In health promotion, contexts include nutrition [35,36,47], physical activity [28,35,47], smoking cessation [48,49], and mental health [38,50].

## Methods

### Overview

In this research, we adopted a constructivist ontological position and acknowledge the socially constructed nature of reality in mHealth systems development [51,52]. Recognizing that *there is no single truth*, constructivist approaches to research generate meaning through a collaborative dialog between researchers and the research participants [51].

### Research Participants

We used a purposive sampling method to identify and recruit participants from 2 groups for interviews, namely, CMEs and MSDs. CMEs were recruited on the web using Google, Google Scholar, LinkedIn, Twitter, and ResearchGate to identify experts in co-design (eg, book authors, academics, and consultants). The MSD group was recruited by searching papers and reports by authors with co-design experience in mHealth. Interviewees had to be aged at least 18 years and fluent in English. Individuals were contacted by the first author via email with a study information statement before obtaining written informed consent. Participation was voluntary and did not involve monetary rewards or other compensation.

Ethics approval was granted by the ethics committee of the University of Newcastle, Australia (H-2019-0064). Data collection and analysis were concurrently performed. The recruitment process continued until data sufficiency was reached (ie, existing categories managed new data without further modifications [53]). The final data set included 16 interviews (8 CMEs and 8 MSDs). On average, CMEs had over 15 years of publication experience (minimum: 2 years; maximum: 25 years) in areas such as co-design or participatory design, cocreation, design thinking, generative design research, and design research methods. On the other hand, MSDs had over 8 years of publication experience on average (minimum: 4 years; maximum: 28 years) in the mHealth literature spanning across multiple areas in disease management (cancer and heart failure) and health promotion (smoking cessation and nutrition). All interviews were audio-recorded (total duration: 14 hours, 15 minutes) and transcribed by the first author. The interview length was between 36 and 72 minutes. Multimedia Appendix 1 provides details on the participants’ backgrounds and experiences.

### Data Collection

Data were collected between July 2019 and January 2020. Before the interview, the research participants received a two-page information statement via email about the research objectives, scheduled interview duration, and assurance of data anonymization. Individuals who provided written consent to participate were interviewed by the first author at a mutually convenient time using Zoom or Skype videoconferencing as per the interviewee’s preference. The interviews were semistructured in nature, with the interviewer using a protocol composed of open-ended questions and probing for additional information when required. The interviews focused on two research objectives: (1) contextualizing an existing co-design framework to the mHealth space and (2) constructing guidelines to address common challenges in this context (see the interview guide in Multimedia Appendix 2). Open-ended questions provided the interviewees with opportunities to speak freely and to guide the discussion in the directions of interest.

### Data Analysis

The first author coded the transcripts following the procedure of Braun and Clarke [15], which included (1) familiarization with the data, (2) coding, (3) searching for themes, (4) reviewing themes, (5) defining and naming themes, and (6) writing up. In step 1, this involved familiarization with the data by repeatedly reading and rereading the transcripts (ie, prolonged engagement). In step 2, the first author performed the initial coding in NVivo. The second author then checked these codes and validated them against the transcripts. Initially, we identified 154 codes from all interviews (eg, power distance and vulnerability). In step 3, the first and second authors clustered nodes into common themes based on coherent patterns. In several discussions between the authors, the identified themes became the foundation of the guidelines. In the results section, data extracts are quoted to support framework contextualization and guideline development. In step 5, the authors further refined the guidelines by eliminating redundant themes and naming the guidelines.

## Results

### Contextualization of Co-design Framework to mHealth Context

**Figure 2:**
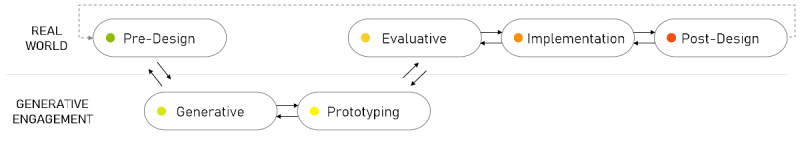
Contextualized co-design framework for mobile health (mHealth).

Figure 2 shows the contextualization of the Sanders and Stappers [13] co-design framework for the mHealth setting. It extends the 4 phases of the original framework by dedicated prototyping and implementation phases. A detailed overview of the example quotes for contextualization is provided in Multimedia Appendix 3.

The first extension was the inclusion of a dedicated *implementation phase*. Interview participants noted that in the context of mHealth, there is a need to separate *implementation* from the evaluative phase. This is because the evaluative phase primarily focuses on testing the *feasibility* of the mHealth system rather than the wider rollout of the system into a complex mHealth ecosystem:

You would not naturally do a clinical trial or a randomized control trial in your implementation phase because you first need to be able to test the feasibility.

MSD8

The implementation phase is after we have done the research and probably after we have analyzed the results and come to some kind of conclusions. So, there is a gap then between the generative phase and the implementation phase when we actually do our research. We are checking to make sure that we have got evidence now that would suggest that this is actually going to support people improve their health outcomes. That is your evaluative phase. Let's now go to the implementation phase where we actually deploy it.

MSD5

In contrast, a dedicated implementation phase should focus on facilitating the integration of mHealth artifacts into complex systems and stakeholder environments. In other words, it is important to not only consider the design of the technical mHealth artifact, but everything else around it that is necessary for it to be successfully implemented. This includes important aspects such as documentation, training, and involving key stakeholders in the rollout (see postdesign advocates, guideline 5). The other important consideration discussed in the interviews was the importance of considering implementation right from the beginning of the co-design process:

You would want implementation to be on the agenda right from the initial co-design process. You need to have a plan. If you are going to co-design something, you need to have a plan that if it is effective, how could it be brought about, and those discussions or those people involved in that process. [...] Having those people involved from the start is fundamental to the success of implementation, and having plans around that.

MSD7

You really need to work with the [health system] and that is where that whole implementation phase becomes crucial because even if your thing is beautiful, if it does not have the support to make it work, it will fall down.

MSD3

The second extension pertains to a separate prototyping phase before the evaluative phase to acknowledge the complexity of mHealth artifacts and their evaluation requirements (eg, pilot-testing and randomized controlled trials). Including a separate prototyping phase assists in separating generative co-design methods in the generative phase (eg, paper prototyping), from instantiations in which the idea for the solution has become more mature and where (high fidelity) prototyping occurs (ie, hardware and software prototypes). Furthermore, it emphasizes the need for a fully functional prototype at the end of the prototyping phase, suitable for rigorous evaluation in the real-world context as part of the evaluative phase (eg, pilot-testing and randomized controlled trials). In addition, separating the prototyping and evaluative phases can clarify which stakeholders should be involved in which phase and in what capacity they should be involved (eg, app developers in the generative phase may simply observe or consult with the end users, whereas in the prototyping phase, they are developing a prototype):

You go from low fidelity [generative], to high fidelity [prototyping], and then to user testing [evaluative]. Generative is like low fidelity brainstorming. Generative design research and making is not user testing. It is different. What you are calling the generative phase is more like wire framing. You increase the fidelity of your prototypes as you go and test along the way.

MSD3

[Initially,] I would not constrain the end-users with any details about what can and cannot be done. [The mHealth system] would be a magic device and they would act out scenarios without any worry about how this could actually be mocked-up. I would have the developers see and hear that and hopefully then be inspired by it to bring somebody's dream to life [...]. But in a later phase you might hand pick some of the end-users to come and work directly with the developers. Then it’s like: ‘Well the end-user’s dreams are this, but the developer's constraints are these. Can you guys come up with something together?

CME1

Finally, it is important to consider the context in which the co-design phases occur. The contextualized framework categorizes the generative and prototyping phases as phases in which *generative engagement* occurs. These phases involve gathering co-design participants from potentially diverse areas (eg, health practitioners and designers) in one place (eg, a co-design workshop in a studio or lab) to engage in generative co-design methods (eg, storyboarding and paper prototyping). However, it is important to note that, especially in the health context, it may not always be possible for end users to gather in the same physical space:

Maybe they cannot get there. Maybe socially it is a challenge for them. Maybe you do engage with those people one-on-one, and then bring things together later. So, an interview, or user testing, or even a digital engagement where you are putting something online and getting some feedback.

CME6

Hence, for co-design to be accessible to end users, generative engagement does not necessarily need to occur in the presence of all stakeholders or in the same physical space. For example, Smeenk et al [54] described an empathic handover approach in which end users can participate in the early phases of co-design alongside a principal designer who later translates these contributions [54]. On the other hand, there are also co-design phases that require immersion in the *real-world* context in which the mHealth system will eventually be implemented (see also guideline 4). For example, in the predesign phase, interviews or observations may be carried out in a hospital to gain a better understanding of the problem and the stakeholders that need to be involved. Given the focus on the real-world context, this immersion is especially important for the predesign, evaluative, implementation, and postdesign phases:

I think a really important part of that was the fact that we were working on-site

MSD1

We position ourselves in the context by submerging ourselves in all the relevant stakeholders

MSD8

### Guidelines for Co-Designing in mHealth

On the basis of the thematic analysis of the interviews, we constructed seven guidelines (guidelines 1-7; Textbox 1) to address the challenges in co-designing mHealth systems. Multimedia Appendix 4 provides a detailed overview of example quotes.

**Guidelines 1-7.**

**Guideline 1**

- Carefully consider the unique circumstances of the targeted disease management or health promotion context with respect to its evaluation and integration requirements, stakeholder involvement, and end user vulnerabilities relating to highly personal aspects of a person’s health.

**Guideline 2**

- As early as possible in the co-design process, consult the behavior change literature and/or involve experts in behavior change relevant to the problem context to effectively identify the targeted change in behavior and adequately plan the type and stakeholder involvement of co-design activities.

**Guideline 3**

- Select and engage co-design facilitators that have an authentic understanding of the intimate problem context (eg, first-hand experience, immersing in problem context, and literature consultation) and operate in an empathetic way to mitigate potential barriers associated with the power distance between mHealth stakeholders.

**Guideline 4**

- Immerse yourself in the underlying complex health context to identify and understand stakeholders early, include them in defining their involvement in the co-design process along existing health process requirements, recognize the diversity and inherent power distances among stakeholders, and prioritize the needs of the end user.

**Guideline 5**

- Throughout every phase of co-design, identify potential postdesign advocates from different stakeholder categories who can aid in implementing the mHealth system (eg, training staff in the use of the system) and champion its use in the postdesign phase (eg, providing feedback on system use in practice).

**Guideline 6**

- In the evaluative phase, ensure that the mHealth system goes through feasibility testing in the real world (pilot-testing and randomized controlled trials) to adequately address ethical considerations in the health context, determine potential risks to the end users caused by the artifact, and clarify whether it accomplishes its intended goals before implementation.

**Guideline 7**

- In the postdesign phase, collect usage data to observe the mHealth system’s impact after it has been implemented and apply contextual co-design methods to understand this impact.

#### Guideline 1: Understanding Stakeholder Vulnerabilities and Diversity

The interviews emphasized important differences between health promotion and disease management, including (1) that mHealth users have unique vulnerabilities, (2) the diverse array of stakeholders involved, (3) the significance of evaluation, and (4) the actual implementation and translation. Owing to the focus on health outcomes, mHealth typically involves vulnerable user groups (users with health conditions that may create additional barriers to participation, eg, patients). Although this vulnerability may be present in some groups within health promotion (eg, smoking cessation and alcohol reduction), it appears to be most prevalent among the disease management cohort (eg, dementia and autism). This vulnerability creates challenges for (1) recruiting representatives from the target cohort and (2) being mindful of their health vulnerabilities:

The first thing that comes to mind [challenge] is getting access to participants. It is really impossible in healthcare. [...] It might be really hard to get people to open up and be honest about their experiences of having a stoma bag [...]. It is just not a subject that ever gets discussed with family members around. [You] can talk to patients one-on-one maybe, but [not] with all their family around them.

CME2

With the specific focus of supporting positive health outcomes, the co-design process inherently touches on vulnerable, deeply personal aspects that, in turn, require high levels of trust in the research team and process. Hence, it is vital to select co-design tools and methods that are appropriate in this context and allow vulnerable end users to participate in the best of their capabilities:

I knew from experience what it was like to be with someone who has this disease, and that made it easier because I already knew the context, because then you know how to behave. I think for people who are not familiar with that, they must be acquainted with that first.

CME3

One [challenge] is a lack of trust. [...] It happens with government led and funded projects where people who may have had a lifetime of being let down by organizations and institutions and they may find it difficult to trust that their voice will really be heard and that things will really change because of their participation.

CME6

Each specific mHealth context also has its own diversity of stakeholders who need to be identified and involved in the co-design process. There may also be more than one category of end users, such as both the patient and the health practitioner, with differing requirements in terms of evaluation:

You have got app designers, [marketing people, health professionals], and you have got the end-users who are trying to grapple with their medical challenges that they have. [...] That would be one of the biggest challenges, getting those people together.

MSD5

The last element of this guideline is the integration of mHealth tools into a wider system landscape. One of the largest identified differences between co-design in mHealth and other contexts is that mHealth systems need to integrate into a highly complex health ecosystem involving an array of health processes, systems, and stakeholders:

It is not just about the end product, it is about everything that goes with it that we need to test and work out too. So, the instructions that we give to people as to how to use it, how we advertise it, who we train in the facility in terms of helping patients to use it, how we promote it to staff so that they know it is available to their patients as well.

MSD1

#### Guideline 2: Planning for and Assessing Health Behavior Change

The second theme refers to the importance of consulting the behavior change literature and considers directly involving behavior change experts in the co-design process. Overall, the importance of consulting the behavior change literature was mentioned in 9 of the interviews (4 CMEs and 5 MSDs). The importance of behavior change for mHealth systems design relates back to the very nature of the underlying health promotion and disease management contexts, in that the purpose of these systems involves some change in user behavior to address a health goal [55]. For health promotion, this commonly refers to a change in lifestyle behaviors, such as reducing alcohol consumption and quitting smoking [49,56] or improving eating habits [47]. For disease management, examples include regularly performing rehabilitation exercises [41], following a specific medication regime [57], or recording specific aspects of daily activities [39,41]. Interviewees emphasized that because behavior change is not a by-product but is integrally linked to the purpose of the mHealth system, it is vital to explore in the early stages of the co-design process which behaviors are addressed and in what way:

You have to engage in the behavior change literature [...]. A health practitioner probably knows that there is behavior change literature to go to, but someone outside that health domain may not know to go to that literature.

MSD8

MSD6 elaborated that a key distinguishing factor between mHealth and other contexts is that co-designing mHealth systems is linked to changes in behavior that are often deeply personal to the end users, which are linked to deeply embedded long-term habits (eg, eating, physical activity, and sleep patterns):

You are talking about changing behaviors that are there for a reason. They are not just trivial behaviors, they are deeply embedded and they have really unusual reasonings that [...] surprise you. Whereas if you are just designing a booking system or whatever, it is not that emotive.

MSD6

Finally, given the focus on mHealth systems to achieve positive health outcomes, it is vital to carefully tailor the co-design activities to the individual circumstances and capabilities of the stakeholders, particularly the end user:

Co-design frameworks [are] very focused on picking a series of methods for a workshop, and then saying, ‘okay participants, I all want you to do this method using these kinds of materials.’ [This] is just completely unfeasible when you have people with only one hand [...]. Co-design [...] for healthcare [...] does have to be approached differently.

CME2

#### Guideline 3: Identifying and Involving Co-design Facilitators

Interviewees emphasized the critical role of facilitators. In mHealth, co-designing involves high stakeholder diversity (eg, app developers, health practitioners, and health insurance providers) while simultaneously addressing highly intimate issues and concerns regarding a person’s health (eg, quitting smoking and diabetes self-management). Against this backdrop, the facilitator plays a critical role in involving stakeholders in a trusted, meaningful, and effective way.

Neglecting the role of the facilitator yields a range of risks, including a lack of true involvement (eg, because of power distance between end users and health professionals) and a lack of understanding about end users’ lived experiences and perspectives:

I think that power balance is particularly interesting in healthcare because it is really hard to say that you do not agree [with] a doctor. [...] They are held up in such high esteem as being experts of the subject matter [...]. So, to then put-up patients in a room [saying] ‘co-design with your doctors’, it could be really confronting to [say] ‘oh I have a different opinion to you and I do not usually get to express it in my experiences with you, but now can I?’

CME2

We were a little bit disconnected from knowing what it truly means to struggle with [an] addiction that you want to give up and you know is bad for you [...]. All these issues are quite emotional and unless you understand how it really feels I think it is important for whoever is running the workshop [to] have a feel for the topic, a knowledge of what it means.

MSD6

To address potential challenges (eg, power distance and lack of empathy), interviewees emphasized that co-design facilitators need an authentic understanding of end users’ real-world experiences (eg, through first-hand experience, immersing in problem context, and consulting relevant literature). Facilitators are then able to operate in a more empathetic way, which can help participants feel more comfortable sharing personal experiences regarding their own health. For example, MSD6 stated that facilitators in their smoking cessation project had personal experience with the context. On the basis of ice-breaking exercises, the authentic experiences of the facilitators enabled them to support stakeholders in becoming more comfortable to actively engage with co-design activities. As a result, the facilitators were perceived as more like co-design participants than authority figures. Empathy, or a “soft human touch” (CME4), is a critical skill for a facilitator running co-design workshops to overcome the inherent power distance issue in the mHealth space:

I think the more practical power distance issues in sessions can be easily navigated if you just have a bit of a soft human touch to ensure that people do not feel like you are the cocky arrogant researcher, expert, designer, or however you are positioning yourself.

CME4

There is comfort that comes from people who are like you. [This] is why I saw the two [facilitators] being so successful with the low self-esteem kids because they themselves started the session talking about their problems. The designers running the session were able to talk about their experiences and how they dealt with it and so then they immediately became not the person leading the co-design activity, but a true co-designer.

MSD6

#### Guideline 4: Immersion Into the mHealth Ecosystem

Co-design involves the effective collaboration of system designers with users and other stakeholders. Frequently raised elements include (1) the importance of being immersed in the context where stakeholders are, for optimal problem identification; (2) identification of relevant stakeholders as early as possible to drive their own involvement and contribute to the study design, including ethics approval; and (3) the need for an ongoing relationship with stakeholders in mHealth that recognizes the power distance between stakeholders and prioritizes the needs of end users.

The multiplicity of factors affecting and supporting a person’s health renders the environment of mHealth system design inherently complex. Interviewees repeatedly stressed the need for system designers to immerse themselves deeply to effectively identify stakeholders, understand pain points and relationships with one another, and correctly determine the problems they can and cannot address:

You do have to be embedded in the space in order to identify it, or you have to be listening to people who are embedded in the space in order to identify it.

MSD1

We started with a empathize phase, [which] was around interviewing all different types of stakeholders individually to try and understand what their experiences are, what their frustrations are, what their behaviors and pain points are, what they really struggle with.

MSD2

Another important aspect relates to involving stakeholders as early as possible because failing to do so is particularly critical, and possibly fatal, in the realm of mHealth. First, because of the array of factors around a person’s health, the number of potential stakeholders is high, which requires buffer times for planning, organizing, communicating, and scheduling. Second, the health sector naturally encompasses complex policies and procedures to adhere to privacy regulations and protect and support vulnerable populations. It is therefore vital for stakeholders to become involved sufficiently early to be able to point out procedural constraints in their domains (eg, requirements and time frames for ethics approvals):

I would say involve them right from the start. [...] Ascertain to what extent they are going to be able to contribute any of their time [...] and ask them what stage they think they want to be involved [...] and let them drive that process.

MSD5

The best way to manage the different stakeholders and the management is at different stages [to] highlight the appropriate stakeholders that are necessary and let them know what their voice is and what their purpose is. Basically, letting stakeholders know when their input is important and needed and what the reason for their input is.

CME8

Support for positive health outcomes is an ongoing process. It follows that the relationship with stakeholders of mHealth systems design needs to be managed and supported in an ongoing way. Although this holds true for both health promotion and disease management, it is particularly critical in the disease management space. It is also critical to balance the number of participants involved in co-design activities and avoid a potential power imbalance geared toward senior medical practitioners. The resulting power distances between the stakeholders must be carefully considered. After all, the person most affected by the system will be the end users and, hence, it is vital to adequately capture and address their needs:

The problem should really be generated in part by the people who are affected when it has something to do with health management. [...] There must be more of an ongoing relationship [with end-users] even if there is not a particular problem yet.

MSD1

I get really concerned when I see just one or two people with lived experience brought on as kind of the token users to a predominantly professional group and you just think how can those people feel confident and comfortable in that setting, especially in a health context where they are used to being told by the professionals.

CME6

#### Guideline 5: Identifying and Involving Postdesign Advocates

Interviewees repeatedly stressed the challenge of implementing and rolling out mHealth systems. This is linked to the complexity and risk of processes in the health sector and the multitude of stakeholders who need to work together effectively. Against this backdrop, we identified the importance of postdesign advocates as an important theme. CME1 described postdesign advocates as “end-users who are really interested in what you are doing, how you are doing it, and what it could mean for them.” MSD7 elaborates that postdesign advocates are the “people behind it that are going to drive, push, and refer patients or their communities to [the mHealth system].” Identifying postdesign advocates is critical for system designers to support the implementation and postdesign of the mHealth system.

Postdesign advocates need to be stakeholders who are well-connected and respected in the application context. By actively involving them early in the co-design process, their contributions can already be considered in the predesign and generative phases. This establishes a “buy-in” of stakeholders that can later assist in championing the system in the implementation and postdesign phases with the people and communities who are going to use the system. In this way, identifying postdesign advocates can mitigate many challenges in mHealth, including integration into clinical practice and collecting data in the postdesign phase:

Implementation of mHealth tools is extraordinarily challenging [...]. There are a lot of barriers of getting things into practice and getting that buy-in from communities [...] can actually aid your implementation because they have already bought in. Because they are invested in it, they are more likely to try and help make it happen.

MSD7

Beyond the technical aspects of the designed system, interviewees argued that the integration into the complex processes and the various stakeholders in the health space renders the implementation and rollout of the system exceptionally challenging. Postdesign advocates can be critical to informing and supporting the implementation of the mHealth system in the real world. If health practitioners do not believe that the system will be useful, they will not promote it to their patients and may actively dissuade use:

If they were not taught how to use [the app] properly, if they were not given the right support materials, or if it did not get to the right people because the people who did the roll out of it were not briefed well enough around the sorts of people we want it to go to, even if it was really beautifully designed, then it would have failed. So, I am talking about the wraparound services of the thing.

MSD3

We talk about champions, you have got to have people behind it that are going to drive it and push it. They are going to refer patients or their communities to it, or they are going to support services to use these tools

MSD7

Finally, another reason for involving postdesign advocates is that stakeholders in practice are essential to measuring the impact of the mHealth system once it has been implemented in the real world (eg, based on usage data) and being available for follow-up co-design activities in the postimplementation phase (eg, postdesign interviews) to make sense of the usage data:

You will find some end-users in this process who are really interested in what you are doing and how you are doing it and what it could mean for them. Those are the kind of people who might become your post-design advocates who would collect this data for you and at a reasonable price because they have a vested interest in seeing how it worked and helping other people manage their lives for example. So, you could build it into the whole process.

CME1

#### Guideline 6: Applying Health-Specific Evaluation Criteria

The evaluation of mHealth systems requires additional considerations compared with other contexts because of the intended and possibly unintended effects of the artifact on people’s health. The main elements uncovered from the interviews were as follows: (1) the risks and ethical issues associated with developing solutions in a health care context and (2) the need for feasibility testing in the real world (eg, clinical trials and pilot-testing), before implementation, to ensure that the mHealth system accomplishes what it set out to do and does not pose a risk to the end users.

Interview participants emphasized that because of the focus on people’s health, there are additional risks and ethical considerations when co-design mHealth systems for a health care context. For example, MSD1 elaborated:

Even though your interruption through technology might end up with things being better, you still have to be very conscious of the fact that there is more at stake if anything goes wrong because I would not want to be involved in a technology that made things more complicated for people who are already in a complicated and stressful situation.

MSD1

To navigate these risks, it is important to ensure that an mHealth system goes through feasibility testing such as pilot-testing and randomized controlled trials in the real world so that it can be established that the mHealth system accomplishes its goals and does not pose a risk to the end users:

You need a randomized controlled trial of the app first, so that is in the evaluation phase, not the implementation phase, to then prove that it increases patient outcomes and then they might adopt it.

MSD2

MSD8 further explained that it is important to understand that there are different levels of feasibility testing that pertain to the quality of the test and the cost to run the test. For example, MSD8 recommended performing pilot-testing before conducting randomized controlled trials for these reasons:

We would never as a health researcher or a health clinician move straight into a randomized controlled trial without pilot data first [...]. In terms of costings, randomized control trials are much more expensive to run and they are the gold star or grade one evidence

MSD8

MSD1 adds that feasibility testing is not only important for ensuring that the mHealth system works and poses no risk to end users. Owing to the extensive costs of upkeep after implementation, finding problems during feasibility testing is beneficial because they can be fixed by re-entering the earlier co-design phases. Thereby, some problems are more likely to be found because testing is performed in a real-world setting:

You need to do it in stages, especially because there is such a massive cost involved in terms of the upkeep of apps [...]. If you can have a prototype, it is not just about testing the prototype, it is also about testing how the prototype works in the real world before you turn it into the end product.

MSD1

#### Guideline 7: Collecting and Analyzing Usage Data to Understand Impact

The final theme focuses on the importance of postdesign. CMEs noted that even though postdesign is important to assess the impact of artifacts in the real world [13], this step is frequently not carried out because of time and cost constraints. However, the postdesign phase is especially important in the mHealth context because impact is driven by changes in health behavior [10], and mHealth systems are intended to be used over extended time spans (eg, diabetes self-management). Hence, mHealth systems must be updated to meet changing user needs:

I think post-implementation and the collection of evidence of the impact of that change is absolutely essential because you are talking about people changing their behavior for better health outcomes”

CME7

All apps need to be updated, and one of the biggest issues with health apps is they are not.

MSD7

In addition, interviewees explained that mHealth is in a unique position to measure this impact because of having access to participant usage data from the mHealth system because of their ability to collect usage data. Thereby, it is important that qualitative co-design methods (eg, postdesign interviews) are used to make sense of this participant usage data:

You have got all these functionality and metrics that you can get from mHealth that you cannot get anywhere else [such as] Google metrics, Google Analytics, and usage statistics [...]. That is a whole avenue of data that you do not have when you do not have mHealth.

MSD8

There are ways to get feedback, like usage statistics. Those do not tell you why. Having more qualitative methods to get feedback is really important.

CME6

## Discussion

### General Discussion

Although extensive research exists on co-design methodology and its general application, limited research has examined the complexities that arise in co-designing mHealth systems. This study aimed to (1) contextualize an existing co-design framework for mHealth and (2) develop guidelines for addressing common challenges. From the 16 interviews conducted with CMEs and MSDs and thematic analysis, we contextualized the co-design framework by Sanders and Stappers [13] to the mHealth context and constructed a set of 7 guidelines.

We identified several important aspects from contextualizing the Sanders and Stappers [13] co-design framework. First, it became apparent that some of the co-design phases should be split up. Although the original framework has an overall generative phase, a separate *prototyping phase* was suggested for mHealth to distinguish between the *generation of early concepts* (eg, low-fidelity prototyping) in the generative phase compared with the *testing of more mature concepts* where maturity is higher (eg, high-fidelity prototyping). Furthermore, a dedicated *implementation phase* distinguishes activities performed during evaluation (eg, pilot-testing and randomized controlled trials) versus implementation (eg, creating documentation, training, and user acceptance). Second, mHealth has its idiosyncrasies regarding the *front-end* of co-design, including the importance of researchers immersing themselves in the complex problem context and diverse stakeholder landscape surrounding a person’s health. Furthermore, this diversity of stakeholders can lead to a power distance issue in the *generative phase.* Therefore, it is important to recognize the vulnerability of mHealth end users and their relationships with other stakeholders that could impede participation. The *evaluative phase* is also affected, as mHealth problems are typically riskier compared with other contexts. Thus, pilot-testing and randomized controlled trials were mentioned by interviewees as suitable evaluation methods for mHealth. Finally, the *postdesign phase* plays a specific role in mHealth because of its intended effects on health behavior. However, this cannot be assessed until the system is deployed. Hence, the postdesign phase is necessary to understand this impact on user behavior and to allow for continued monitoring and maintenance.

**Figure 3:**
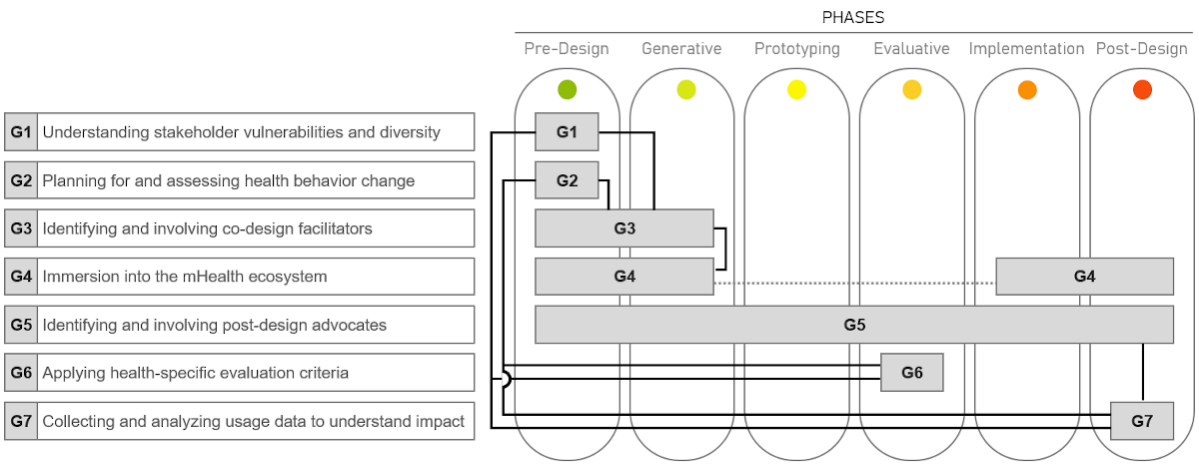
Interplay of guidelines and mapping to the co-design phases.

Addressing the second research objective, seven guidelines were synthesized for applying co-design to mHealth (labeled guideline 1 to guideline 7). As shown in Figure 3, the guidelines pertain to the specific phases of the co-design process. Emphasizing the importance of the front end of co-design, guideline 1 to guideline 4 focus on ensuring that researchers and practitioners establish an intimate understanding of the problem context as early as possible. Interviewees noted that, by following these steps, common challenges such as stakeholder identification, power distance, and lack of trust can be addressed effectively. For instance, by immersing oneself in the mHealth problem context (guideline 4), researchers and practitioners can better understand how end users interface with stakeholders in their health ecosystem, aiding in stakeholder identification. Guideline 5 maps to all phases in the framework as (postdesign) advocates (ie, users championing the system) can be identified in any phase. Interviewees noted that these advocates can help mitigate many issues that can potentially surface in the implementation phase, for instance, by championing the system themselves and by training others. Next, guideline 6 maps to the evaluative phase and emphasizes the importance of health-specific evaluation (eg, pilot-testing and randomized controlled trials) given the high-risk nature of mHealth challenges. Finally, guideline 7 maps to the postdesign phase to ensure that the impact is measured post implementation along with contextual research that informs further system refinements.

Beyond phase-specific relevance, there is also an important interplay between the guidelines. First, guidelines 1, 2, and 4 are linked to 3 because a deep *understanding* of the problem context is needed to effectively apply guideline 3 (highlighting the importance of the front-end of co-design in mHealth). Without this, many of the challenges identified by the interviewees (eg, power distance, lack of trust, and accessibility of tools and methods) would compromise later co-design phases. Second, there is a link between guideline 1/guideline 2 and guideline 6/guideline 7. Guideline 1 and 2 primarily focus on understanding the problem context and establishing the desired goals of the mHealth system, while guidelines 6 and 7 refer to the evaluation of how well the mHealth system addresses these goals, both pre- and postimplementation. Finally, there is a link between guidelines 5 and 7 since the identified advocates will invariably be needed to understand the impact of the mHealth system in the real world.

There were key differences and similarities between the responses of CMEs and MSDs, with implications for the results presented in this paper. First, the interview guide acknowledged the *differing nature of expertise* between the CME and MSD groups to elucidate the experts’ specific domain knowledge (Multimedia Appendix 2). For instance, questions for CMEs primarily referred to co-design in a *general* sense, which tapped into their expertise in co-design applications, processes, phases, methods, and tools. Conversely, questions for MSDs focused on how co-design manifested in their own projects, which led to more *specific* responses about the *contextualization of co-design to mHealth* (challenges they had faced, how they involved mHealth stakeholders, benefit of co-design to their project, etc). The groups expressed similar considerations around the challenges and benefit of co-design, since cost and time constraints are typically common factors in co-design processes, regardless of the context. There was general between-group consensus regarding the aspects that would inform the derivation of guidelines and the contextualization of the framework. Overall, the responses from CMEs tended to refer to general considerations around applying co-design to a complex area, whereas the responses from MSDs were more specific to challenges and best practices based on experience from actual mHealth projects.

### Implications

This work has several important implications for researchers and practitioners. First, building on the extensive expertise of CMEs and MSDs who participated in this research, the contextualized framework may provide a shared frame of reference to guide mHealth systems development projects, which are interdisciplinary in nature [5,6]. Rooted in the widely used co-design framework by Sanders and Stappers [13], the contextualized framework brings to light a range of critical considerations that arise in the health context. As a shared frame of reference, the contextualized framework may aid mHealth researchers and practitioners in planning co-design activities and involving stakeholders in all stages of design [1,6]:

This is great work. There is definitely work in what you are doing. [...] Any type of framework that helps us to do this on the ground more effectively and in [the mHealth] context, that is the kind of work that we need.

CME4

Complementary to the framework, the guidelines point to pitfalls in mHealth systems development along with specific suggestions on how these challenges can be navigated. Multimedia Appendix 5 provides a checklist for co-designing mHealth systems projects according to the 7 guidelines. By facilitating stakeholder engagement and involvement in co-design activities, these guidelines may help researchers and practitioners to ensure that mHealth systems are underpinned by expert insight, reflect the lived experiences of end users, and integrate into the existing system and process landscape [5,11]. In so doing, co-designed mHealth artifacts may enable end users and health professionals to develop a stronger sense of ownership and agency over the outcome. Researchers and practitioners can actively engage postdesign advocates to assist in increasing buy-in from stakeholders, overcoming barriers, and championing the system’s implementation and use.

### Limitations and Opportunities for Future Research

This study had some limitations. First, it should be noted that most of the interviewees resided in the Oceania region and/or were working within the academic sector. Future research may bring to light potential differences in co-design between industry and academia as well as geographical differences related to cultural factors (eg, uncertainty avoidance and power distance [58]). Second, while our research builds on the expertise of the interviewed experts beyond the scope of an individual mHealth system, future research is warranted on the usefulness of the contextualized framework and guidelines for the development of actual mHealth systems. Importantly, this evaluation and refinement should also include the perspectives and lived experiences of individuals involved as co-designers in the context of a specific mHealth system. This was beyond the scope of this study, as we considered mHealth system design beyond the scope of a specific mHealth systems development project. Third, because the current focus is on the co-design process as a whole, it was beyond the scope of this study to assess the applicability of specific co-design methods for mHealth (eg, cultural probes and journey maps). Future research needs to investigate the usefulness and boundary conditions of individual co-design methods in mHealth. This would have the potential to illuminate specific activities that can assist in stakeholder engagement and impact determination in the long run.

### Conclusions

With the focus of supporting positive health outcomes, researchers and practitioners encounter unique challenges in mHealth systems development. Following a constructivist approach, we interviewed 16 experts in co-design methods and mHealth systems development to contextualize an established co-design framework for the mHealth setting and to construct a set of tangible guidelines to address common challenges in this space. While contextualization emphasizes the need to include dedicated prototyping and implementation phases, the guidelines provide practical insights on how to engage in this process by (1) understanding stakeholder vulnerabilities and diversity, (2) planning for and assessing health behavior change, (3) identifying co-design facilitators, (4) immersing in the mHealth ecosystem, (5) identifying postdesign advocates, (6) applying health-specific evaluation criteria, and (7) analyzing usage data and contextual research to understand impact. We hope that the contextualized framework and guidelines presented in this work will serve as a shared frame of reference to facilitate interdisciplinary collaboration at the nexus of information technology and health research.

## Appendix

Multimedia Appendix 1 Background of interview participants.

Multimedia Appendix 2 Interview guide.

Multimedia Appendix 3 Example quotes for framework contextualization.

Multimedia Appendix 4 Example quotes for the 7 guidelines.

Multimedia Appendix 5 Checklist for system designers.

## Abbreviations

CME: co-design method expert
IT: information technology
mHealth: mobile health
MSD: mHealth system designer

## References

[1] Eckman M, Gorski I, Mehta K (2016). Leveraging design thinking to build sustainable mobile health systems. J Med Eng Technol.

[2] Wowak KD, Adjerid I, Angst CM, Guzman JC (2016). A tutorial on empirical ICT4D research in developing countries: processes, challenges, and lessons. Commun Assoc Inf Syst.

[3] (2011). mHealth: new horizons for health through mobile technologies. World Health Organization.

[4] (2018). The rise of mHealth apps: a market snapshot. Liquid State.

[5] Burke LE, Ma J, Azar KM, Bennett GG, Peterson ED, Zheng Y, Riley W, Stephens J, Shah SH, Suffoletto B, Turan TN, Spring B, Steinberger J, Quinn CC (2015). Current science on consumer use of mobile health for cardiovascular disease prevention. Circulation.

[6] Marzano L, Bardill A, Fields B, Herd K, Veale D, Grey N, Moran P (2015). The application of mHealth to mental health: opportunities and challenges. Lancet Psychiatry.

[7] Winters N, Oliver M, Langer L (2017). Can mobile health training meet the challenge of 'measuring better'?. Comp Educ.

[8] Sanders EB, Stappers PJ (2008). Co-creation and the new landscapes of design. CoDesign.

[9] Medhanyie A, Moser A, Spigt M, Yebyo H, Little A, Dinant G, Blanco R (2015). Mobile health data collection at primary health care in ethiopia: a feasible challenge. J Clin Epidemiol.

[10] Noorbergen TJ, Adam MT, Attia JR, Cornforth DJ, Minichiello M (2019). Exploring the design of mHealth systems for health behavior change using mobile biosensors. Commun Assoc Inf Syst.

[11] Moller AC, Merchant G, Conroy DE, West R, Hekler E, Kugler KC, Michie S (2017). Applying and advancing behavior change theories and techniques in the context of a digital health revolution: proposals for more effectively realizing untapped potential. J Behav Med.

[12] Garnett C, Crane D, Michie S, West R, Brown J (2016). Evaluating the effectiveness of a smartphone app to reduce excessive alcohol consumption: protocol for a factorial randomised control trial. BMC Public Health.

[13] Sanders EB, Stappers PJ (2014). Probes, toolkits and prototypes: three approaches to making in codesigning. CoDesign.

[14] Visser FS, Stappers PJ, van der Lugt R, Sanders EB (2005). Contextmapping: experiences from practice. CoDesign.

[15] Braun V, Clarke V (2006). Using thematic analysis in psychology. Qual Res Psychol.

[16] Danaher BG, Brendryen H, Seeley JR, Tyler MS, Woolley T (2015). From black box to toolbox: outlining device functionality, engagement activities, and the pervasive information architecture of mHealth interventions. Internet Interv.

[17] O'Reilly GA, Spruijt-Metz D (2013). Current mHealth technologies for physical activity assessment and promotion. Am J Prev Med.

[18] Kitsiou S, Paré G, Jaana M, Gerber B (2017). Effectiveness of mHealth interventions for patients with diabetes: an overview of systematic reviews. PLoS One.

[19] Ho SY, Guo X, Vogel D (2019). Opportunities and challenges in healthcare information systems research: caring for patients with chronic conditions. Commun Assoc Inf Syst.

[20] McCurdie T, Taneva S, Casselman M, Yeung M, McDaniel C, Ho W, Cafazzo J (2012). mHealth consumer apps: the case for user-centered design. Biomed Instrum Technol.

[21] Jang H, Han SH, Kim JH (2020). User perspectives on blockchain technology: user-centered evaluation and design strategies for dapps. IEEE Access.

[22] Banos O, Villalonga C, Garcia R, Saez A, Damas M, Holgado-Terriza JA, Lee S, Pomares H, Rojas I (2015). Design, implementation and validation of a novel open framework for agile development of mobile health applications. Biomed Eng Online.

[23] Schnall R, Rojas M, Bakken S, Brown W, Carballo-Dieguez A, Carry M, Gelaude D, Mosley JP, Travers J (2016). A user-centered model for designing consumer mobile health (mHealth) applications (apps). J Biomed Inform.

[24] Nahum-Shani I, Smith SN, Spring BJ, Collins LM, Witkiewitz K, Tewari A, Murphy SA (2018). Just-in-time adaptive interventions (JITAIs) in mobile health: key components and design principles for ongoing health behavior support. Ann Behav Med.

[25] Brandt E, Binder T, Sanders E (2012). Tools and techniques: ways to engage telling, making and enacting. Routledge International Handbook of Participatory Design.

[26] Eyles H, Jull A, Dobson R, Firestone R, Whittaker R, Te Morenga L, Goodwin D, Mhurchu CN (2016). Co-design of mHealth delivered interventions: a systematic review to assess key methods and processes. Curr Nutr Rep.

[27] Noorbergen TJ, Adam MT, Roxburgh M, Teubner T (2021). Co-design in mHealth systems development: insights from a systematic literature review. AIS Trans Human-Computer Interact.

[28] Capel T, Schnittert J, Snow S, Vyas D (2015). Exploring motivations of young adults to participate in physical activities. Proceedings of the 33rd Annual ACM Conference Extended Abstracts on Human Factors in Computing Systems.

[29] Danbjørg DB, Villadsen A, Gill E, Rothmann MJ, Clemensen J (2018). Usage of an exercise app in the care for people with osteoarthritis: user-driven exploratory study. JMIR mHealth uHealth.

[30] Kanstrup A (2014). Design concepts for digital diabetes practice: design to explore, share, and camouflage chronic illness. Int J Des.

[31] Wechsler J (2015). HCD mobile health project: post collaboration reflection of researcher and designer. Proceedings of the Asia Pacific HCI and UX Design Symposium.

[32] Aljaroodi H, Adam M, Chiong R, Cornforth D, Minichiello M (2017). Empathic avatars in stroke rehabilitation: a co-designed mHealth artifact for stroke survivors. Proceedings of the International Conference on Design Science Research in Information System and Technology.

[33] Cordova D, Bauermeister JA, Fessler K, Delva J, Nelson A, Nurenberg R, Mendoza Lua F, Alers-Rojas F, Salas-Wright CP, Youth Leadership Council (2015). A community-engaged approach to developing an mHealth HIV/STI and drug abuse preventive intervention for primary care: a qualitative study. JMIR mHealth uHealth.

[34] Das A, Bøthun S, Reitan J, Dahl Y (2015). The use of generative techniques in co-design of mHealth technology and healthcare services for COPD patients. Proceedings of the International Conference of Design, User Experience, and Usability.

[35] Te Morenga L, Pekepo C, Corrigan C, Matoe L, Mules R, Goodwin D, Dymus J, Tunks M, Grey J, Humphrey G, Jull A, Whittaker R, Verbiest M, Firestone R, Ni Mhurchu C (2018). Co-designing an mHealth tool in the New Zealand Māori community with a "Kaupapa Māori" approach. AlterNative.

[36] Verbiest ME, Corrigan C, Dalhousie S, Firestone R, Funaki T, Goodwin D, Grey J, Henry A, Humphrey G, Jull A, Vano M, Pekepo C, Morenga LT, Whittaker R, Mhurchu CN (2019). Using codesign to develop a culturally tailored, behavior change mHealth intervention for indigenous and other priority communities: a case study in New Zealand. Transl Behav Med.

[37] De la Harpe R (2012). Lessons learnt from the participatory design of a mobile care data application in a resource-restricted context. Proceedings of the 12th Participatory Design Conference: Exploratory Papers, Workshop Descriptions, Industry Cases.

[38] VanHeerwaarden N, Ferguson G, Abi-Jaoude A, Johnson A, Hollenberg E, Chaim G, Cleverley K, Eysenbach G, Henderson J, Levinson A, Robb J, Sharpe S, Voineskos A, Wiljer D (2018). The optimization of an eHealth solution (Thought Spot) with transition-aged youth in postsecondary settings: participatory design research. J Med Internet Res.

[39] Castensøe-Seidenfaden P, Reventlov Husted G, Teilmann G, Hommel E, Olsen BS, Kensing F (2017). Designing a self-management app for young people with type 1 diabetes: methodological challenges, experiences, and recommendations. JMIR mHealth uHealth.

[40] Lipson-Smith R, White F, White A, Serong L, Cooper G, Price-Bell G, Hyatt A (2019). Co-design of a consultation audio-recording mobile app for people with cancer: The secondears app. JMIR Form Res.

[41] Davis SR, Peters D, Calvo RA, Sawyer SM, Foster JM, Smith L (2018). "Kiss myAsthma": using a participatory design approach to develop a self-management app with young people with asthma. J Asthma.

[42] Peters D, Davis S, Calvo RA, Sawyer SM, Smith L, Foster JM (2017). Young people's preferences for an asthma self-management app highlight psychological needs: a participatory study. J Med Internet Res.

[43] Woods L, Cummings E, Duff J, Walker K (2017). The development and use of personas in a user-centred mHealth design project. Proceedings of the 29th Australian Conference on Computer-Human Interaction.

[44] Woods L, Cummings E, Duff J, Walker K (2018). Conceptual design and iterative development of a mHealth app by clinicians, patients and their families. Stud Health Technol Inform.

[45] Woods L, Cummings E, Duff J, Walker K (2017). Design thinking for mHealth application co-design to support heart failure self-management. Stud Health Technol Inform.

[46] Løventoft P, Nørregaard L, Frøkjær E (2012). Designing daybuilder: an experimental app to support people with depression. Proceedings of the 12th Participatory Design Conference: Exploratory Papers, Workshop Descriptions, Industry Cases.

[47] Dol A, Kulyk O, Velthuijsen H, van Gemert-Pijnen L, van Strien T (2016). Denk je zèlf! Developing a personalised virtual coach for emotional eaters using personas. Proceedings of the The Eighth International Conference on eHealth, Telemedicine, and Social Medicine (eTelemed 2016).

[48] Paay J, Kjeldskov J, Skov M, Lichon L, Rasmussen S (2015). Understanding individual differences for tailored smoking cessation apps. Proceedings of the 33rd Annual ACM Conference on Human Factors in Computing Systems.

[49] Paay J, Kjeldskov J, Brinthaparan U, Lichon L, Rasmussen S, Srikandaraja N (2017). QuittyLink: Involving smokers in the design of technology that supports individuals in quitting. Proceedings of the Fourth International Conference on Design4Health 2017.

[50] Arslan P, Nam H, Romero M, Perego P, Costa F, Andreoni G, Muschiato S (2010). MOHE: Mobile health for moms, kids, adults and elderly. Proceedings of the First International Conference on Design Creativity, ICDC 2010.

[51] Mann K, MacLeod A, Cleland J, Durning SJ (2015). Constructivism: learning theories and approaches to research. Researching Medical Education.

[52] Wachtler C, Brorsson A, Troein M (2006). Meeting and treating cultural difference in primary care: a qualitative interview study. Fam Pract.

[53] Varpio L, Ajjawi R, Monrouxe LV, O'Brien BC, Rees C (2017). Shedding the cobra effect: problematising thematic emergence, triangulation, saturation and member checking. Med Educ.

[54] Smeenk W, Sturm J, Eggen B (2018). Empathic handover: how would you feel? Handing over dementia experiences and feelings in empathic co-design. CoDesign.

[55] Free C, Phillips G, Galli L, Watson L, Felix L, Edwards P, Patel V, Haines A (2013). The effectiveness of mobile-health technology-based health behaviour change or disease management interventions for health care consumers: a systematic review. PLoS Med.

[56] Gustafson DH, McTavish FM, Chih M, Atwood AK, Johnson RA, Boyle MG, Levy MS, Driscoll H, Chisholm SM, Dillenburg L, Isham A, Shah D (2014). A smartphone application to support recovery from alcoholism: a randomized clinical trial. JAMA Psychiatry.

[57] Vilarinho T, Floch J, Stav E (2017). Co-designing a mHealth application for self-management of cystic fibrosis. Proceedings of the IFIP Conference on Human-Computer Interaction.

[58] Matusitz J, Musambira G (2013). Power distance, uncertainty avoidance, and technology: analyzing hofstede's dimensions and human development indicators. J Technol Hum Serv.

