# Supplementary material for: Using Co-design in Mobile Health System Development: A Qualitative Study With Experts in Co-design and Mobile Health System Development
Source: JMIR Mhealth Uhealth. 2021 Nov 10;9(11):e27896. doi: 10.2196/27896 (PMC8663505; doi:10.2196/27896)
Supplement: Multimedia Appendix 1 [file mhealth_v9i11e27896_app1.docx]

## Multimedia Appendix 1: Interview Participants

**Table 1.** Background of interview participants

| **Stakeholder** | **ID** | **Background and Experience** | **Region** | **Gender** |
| --- | --- | --- | --- | --- |
| Co-design  Method  Expert (CME) | CME1 | Academic. PhD, M.A, B.A. 25+ years publication experience in design. 10+ years of experience as a design research consultant. | North America | Female |
|  | CME2 | UX designer at a private company with more than 2 years of experience. MPhil (Design), B.A and B.Des (Hons). | Oceania | Female |
|  | CME3 | Academic. PhD. 12+ years publication experience in design. 10+ years working as a design professional in industry. | Europe | Female |
|  | CME4 | Academic. PhD. 9+ years publication experience in the field of design with over 100 publications. | Oceania | Male |
|  | CME5 | Academic. PhD, MSc. 19+ year publication experience in design. | Europe | Female |
|  | CME6 | Freelance design research consultant with more than 4 years of experience. PhD. 8 years publication experience in design. | Oceania | Female |
|  | CME7 | Academic. PhD. 25 years of publication experience in design. | Oceania | Male |
|  | CME8 | Academic. PhD. 12 years of publication experience in design. | Oceania | Female |
| mHealth System Developers (MSD) | MSD1 | Academic. PhD. B.A, BSc(hons). 6 years publication experience in mHealth. | Oceania | Female |
|  | MSD2 | Academic. PhD. 4 years of publication experience in mHealth. 10 years of experience in healthcare industry. | Oceania | Female |
|  | MSD3 | UX designer at a private company with 15 years of experience in industry. M.Des, B.A. Has publication experience in mHealth. | Oceania | Female |
|  | MSD4 | Academic. PhD, M.A, B.A. 20+ years of publication experience in design and health with 100+ publications. | Europe | Female |
|  | MSD5 | Academic. PhD, BSc. 28 years of publication experience overall. 3+ years of publication experience in mHealth. | Oceania | Male |
|  | MSD6 | Academic. PhD. 24 years of publication experience overall. 7 years of publication experience in mHealth. | Oceania | Female |
|  | MSD7 | Academic. PhD, MSc. 12 years of publication experience overall. 4 years of publication experience in mHealth | Oceania | Female |
|  | MSD8 | Academic. PhD, BNutrDiet (Hons). 15 years of publication experience with 150+ publications. | Oceania | Female |
| *Note:*  B.A = Bachelor of Arts, B.Des = Bachelor of Design, BNutrDiet = Bachelor of Nutrition and Dietetics, BSc = Bachelor of Science, M.A = Master of Arts, M.Des = Master of Design, MPhil = Master of Philosophy, (Hons) = Honours, MSc = Master of Science | | | | |
