# Supplementary material for: Using Co-design in Mobile Health System Development: A Qualitative Study With Experts in Co-design and Mobile Health System Development
Source: JMIR Mhealth Uhealth. 2021 Nov 10;9(11):e27896. doi: 10.2196/27896 (PMC8663505; doi:10.2196/27896)
Supplement: Multimedia Appendix 2 [file mhealth_v9i11e27896_app2.docx]

## Multimedia Appendix 2: Interview Guide

The semi-structured interviews in this research were guided by the co-design framework by Sanders and Stappers [22], which was iteratively contextualized throughout the interviews (i.e., from Figure 1 to Figure 2 in the paper). Questions were structured around the two overarching research objectives, namely (1) contextualizing the existing co-design framework by Sanders and Stappers [22] to mHealth and (2) deriving guidelines suitable to overcome common challenges of using co-design in mHealth development. Through the interviews, the researcher attempted to expand and refine this contextualized framework by uncovering emergent and additional themes as well as furthering the understanding of existing themes. Questioning was of a general nature and semi-open ended in the context of co-design and mHealth system design. More specifically, the focus of the interviews was to solicit responses, insights, and suggestions regarding the following areas:

- The value of using co-design in mHealth systems development
- The contribution of co-design to mHealth systems development
- The disadvantages/risks of using co-design in mHealth systems development
- The value added by co-design in specific phases
- Lessons to be learned when using co-design in mHealth systems development
- The use and misuse of the term co-design in research and practice

Examples of Questions for Co-design Method Experts (CMEs)

- From our literature search we found that co-design has recently receive increased research attention. Based on your expertise and reading of the literature, do you feel that co-design is adequately applied in practice?
- Based on your expertise, do you feel that the term co-design is adequately used in research and practice? Is the term potentially misused to label research co-design that would not be truly classified as co-design based on your expertise?
- What do you think would be the barriers and challenges of applying co-design in a mHealth context? What advice would you give to researchers for overcoming these barriers and challenges?
- In the application of co-design, are there specific methods that are underused or misused?
- Do you think that co-design is adequately applied across the whole design process or are there certain aspects over/underrepresented?

Examples of Questions for mHealth System Developers (MSDs)

- Why did you choose to employ co-design in your study?
- What were the co-design phases you went through in your study? What is the purpose of each phase? Does the level of involvement with stakeholders differ from phase to phase?
- What was your rationale for the specific methods you chose to employ in your study? What co-design phase were these methods used in and why?
- What was the added value of using co-design in your study?
- What were the barriers and challenges of using co-design in your study?
- How did you evaluate your designs and when did this occur?
- What advice would you give to mHealth system developers that are looking to use co-design in their project?
