# Supplementary material for: Using Co-design in Mobile Health System Development: A Qualitative Study With Experts in Co-design and Mobile Health System Development
Source: JMIR Mhealth Uhealth. 2021 Nov 10;9(11):e27896. doi: 10.2196/27896 (PMC8663505; doi:10.2196/27896)
Supplement: Multimedia Appendix 3 [file mhealth_v9i11e27896_app3.docx]

## Multimedia Appendix 3: Example Quotes for Framework Contextualization

**Table 1.** Example quotes for the contextualization of the co-design framework

|  | **Contextualization to mHealth** | **Example Quote from Interviews** |
| --- | --- | --- |
| **Pre-Design Phase** | Unpacking of the person’s complex health ecosystem | “[…] part of the process in the pre-design phase is unpacking the ecosystem for the person. Who are the organizations they interface with relating to their health? Who are the people that they interface with related to their health? That helps you build up a bigger picture and where the leverage points for change are.” **[MSD3]** |
|  | Researcher immersion into the context the mHealth system resides in | “I think a really important part of that was the fact that we were working on-site” **[MSD1]**  “[…] we position ourselves in the context by submerging ourselves in all the relevant stakeholders” **[MSD8]** |
| **Generative Phase** | Vulnerability of end-users in the mHealth context requires involvement in co-design activities to be as safe and accessible as possible | “It is about getting people to participate on the level that they are comfortable with […]. Sometimes that means it is not actually appropriate or ideal to bring a diverse group together because that might not be safe, fun, or easy […]. Sometimes [you] might need to do work with participants in advance of bringing a diverse group together to make sure that every single person there understands what they can do [to be as inclusive as possible].” **[CME6]** |
|  | Diversity of mHealth stakeholders requires low fidelity prototypes to be sufficiently simplistic to engage with and provide feedback | “[It] was really important that it [prototype] was not anything too complex or new software. I developed the wireframes and went back to every team member, so those that participated in the design workshops, but also others that did not want to come. They were too intimidated to come to the design workshops. I met with them one-on-one, had a coffee with them, and showed them the PowerPoint so that they could comment and improve on the design.” **[MSD2]** |
| **Prototyping Phase*** | Separation of prototyping in the generative phase (e.g., paper prototyping) from solution instantiations (e.g., high fidelity prototypes) due to complexity of mHealth systems | “Even design professionals themselves are sometimes reluctant to do really low-fi prototyping. I think people often feel if we are making something, we need to make it really high quality and show people how good it is going to be. But there is real value in making stuff with paper, or plasticine […] in the health context. I have seen some good examples of people using little figurines and models of how people interact in places like emergency rooms […] and acting it out […]. What is a cheap and fast way to test out some of these ideas? […] What are the questions we need to answer to know what we need to move on to the next phase and actually start designing or developing a fuller scale higher fidelity solution.” **[MSD6]**  “You have to have a pilot test of it for feasibility of deploying it in the real world because there is going to be quite a difference between what you have generated in the generative phase and what can actually be taken up day-to-day in the real world […]. You need to do it in stages, especially because there is such a massive cost involved in terms of the upkeep of apps and other technologies too. So if you can have a prototype, it is not just about testing the prototype, it is also about testing how the prototype works in the real world before you turn it into the end product.” **[MSD1]**  “You go from low fidelity [generative], to high fidelity [prototyping], and then to user testing [evaluative]. Generative is like low fidelity brainstorming. Generative design research and making is not user testing. It is different. What you are calling the generative phase is more like wire framing. You increase the fidelity of your prototypes as you go and test along the way. That is key for this.” **[MSD3]**  “[Initially,] I would not constrain the end-users with any details about what can and cannot be done. [The mHealth system] would be a magic device and they would act out scenarios without any worry about how this could actually be mocked-up. I would have the developers see and hear that and hopefully then be inspired by it to bring somebody's dream to life […]. But in a later phase you might hand pick some of the end-users to come and work directly with the developers. Then it’s like: ‘Well the end-user’s dreams are this, but the developer's constraints are these. Can you guys come up with something together?’” **[CME1]** |
| **Evaluative Phase** | Health-specific evaluation requirements due to impact on people’s health (e.g., pilots, randomized control trials) | “You would not naturally do a clinical trial or a randomized control trial in your implementation phase because you first need to be able to test the feasibility. So, you would not do […] the randomized control trial until you have got some pilot data back and done it. I would definitely split that up because they are pretty high-level kind of quality study designs, so you would only save that for once you have got some pilot data. We would never as a health researcher or a health clinician move straight into a randomized controlled trial without pilot data first […]. In terms of costings, randomized control trials are much more expensive to run and they are the gold star or grade one evidence.” **[MSD8]**  “The implementation phase is after we have done the research and probably after we have analyzed the results and come to some kind of conclusions. So, there is a gap then between the generative phase and the implementation phase when we actually do our research. We are checking to make sure that we have got evidence now that would suggest that this is actually going to support people improve their health outcomes. That is your evaluative phase. Let's now go to the implementation phase where we actually deploy it.” **[MSD5]** |
| **Implementation Phase*** | Training and supportive materials for end-users and health practitioners to seamlessly integrate the mHealth system into the complex process and system landscape | “It is not just about the end-product, it is about everything that goes with it that we need to test and work out too. So the instructions that we give to people as to how to use it, how we advertise it, who we train in the facility in terms of helping patients to use it, how we promote it to staff so that they know it is available to their patients as well. So all of that is about the implementation, and I think the implementation needs a generative phase as well as the development of the mHealth system needing a generative phase, and maybe that generative phase is pilot testing it in some way.” **[MSD1]**  “If they were not taught how to use [the app] properly, if they were not given the right support materials, or if it did not get to the right people because the people who did the roll out of it were not briefed well enough around the sorts of people we want it to go to, even if it was really beautifully designed, then it would have failed. So, I am talking about the wraparound services of the thing. It is not just a thing.” **[MSD3]** |
|  | Complexity of processes and systems in the health context requires the implementation to be a consideration right from the start of the co-design process (who and what is involved to be successful) | “You really need to work with the [health system] and that is where that whole implementation phase becomes crucial because even if your thing is beautiful, if they do not have the support to make it work, it will fall down.” **[MSD3]**  “So typically, you would want implementation to be on the agenda right from the initial co-design process. You need to have a plan. If you are going to co-design something, you need to have a plan that if it is effective, how could it be brought about, and those discussions or those people involved in that process. Even when you do that, the co-design process is hugely lengthy, typically. It is very hard to do that quickly. And then also the research process: if you are going to do robust research, that is extremely lengthy. By the time you actually get to a position where you might be looking at implementation, the climate can have changed drastically. Having those people involved from the start is fundamental to the success of implementation, and having plans around that.” **[MSD7]** |
| **Post-Design Phase** | With the focus of mHealth systems on user health behavior, it is essential to collect and analyze post-design usage data | “I think post-implementation and the collection of evidence of the impact of that change is absolutely essential because you are talking about people changing their behavior for better health outcomes.” **[CME7]**  “In this post-implementation phase […] you can get post-design interviews and scenarios, but the additional part of that is that you have got all these functionality and metrics that you can get from mHealth that you cannot get anywhere else. So, like Google metrics, Google Analytics, and usage statistics and stuff like that. That is a whole avenue of data that you do not have when you do not have mHealth.” **[MSD8]** |

***Note:*** * = extension beyond the original Sanders and Stappers framework
