# Supplementary material for: Using Co-design in Mobile Health System Development: A Qualitative Study With Experts in Co-design and Mobile Health System Development
Source: JMIR Mhealth Uhealth. 2021 Nov 10;9(11):e27896. doi: 10.2196/27896 (PMC8663505; doi:10.2196/27896)
Supplement: Multimedia Appendix 4 [file mhealth_v9i11e27896_app4.docx]

## Multimedia Appendix 4: Example Quotes for the Seven Guidelines

**Table 1.** Example quotes for the seven guidelines

| **Guideline** | **Example Quote from Interviews** |
| --- | --- |
| **Guideline 1:**  Understanding stakeholder vulnerabilities and diversity | “The first thing that comes to mind [challenge] is getting access to participants. It is really impossible in healthcare. […] It might be really hard to get people to open up and be honest about their experiences of having a stoma bag […]. It could be that [you] think a subject is not sensitive, but then in the case of another culture […]. It is just not a subject that ever gets discussed with family members around. [You] can talk to patients one-on-one maybe, but [not] with all their family around them. I suppose there could be really interesting cultural sensitivities or just general health sensitivities.” **[CME2]**  “Especially when we are talking about long-term conditions […], there were high numbers of [indigenous] people who were involved and needed to be involved, and that is just one example of how you have got to think about what the cultural norms are too in terms of participating […]. Especially when we had projects that were around young people […], young people who were following cultural norms would never contradict or talk before an elder […]. That is also something to be mindful of. What are the cultural norms of the people that will affect their participation?” **[CME6]**  “If you go to critical situations like people with dementia or autism […] it is very important to have a designer or a researcher that has specific characteristics or traits […]. You also must be emotionally interested in your users and especially with the people that are more vulnerable or sensitive […]. I knew from experience what it was like to be with someone who has this disease, and that made it easier because I already knew the context, because then you know how to behave. I think for people who are not familiar with that, they must be acquainted with that first […]. You need to do that if you do not have this experience already before you can start thinking of which methods to use and how to set up your design process.” [CME3]  “One [challenge] is a lack of trust. [It] might be that you are being invited to participate in a co-design process and you do not trust the person or organization to actually [really] listen to you. It happens with government led and funded projects where people who may have had a lifetime of being let down by organizations and institutions and they may find it difficult to trust that their voice will really be heard and that things will really change because of their participation.” **[CME6]**  “[As] soon as you bring a group together [there] needs to be trust and disclosure happening. [One] of the people said to us afterwards, ‘I have all this personal stuff I did not want to talk about it in the group. So, I didn’t say anything.’ [There] is that aspect of the deeply personal secrets […] because we talk about behavior change and […] resisting that change. [Otherwise] you would not have your app. […] They have to be really honest about why they cannot and a lot of people do not want to be honest about that and not in a group situation.” **[MSD6]**  “You have got app designers, [marketing people, health professionals], and you have got the end-users who are trying to grapple with their medical challenges that they have. […] That would be one of the biggest challenges, getting those people together. […] You might get people with illnesses that have a bit of time on their hands and welcome a bit of attention to their condition and something that might help them. On the other hand, you have got clinicians who are massively busy and would not want to spend more than five minutes on this. […] There will be other people who would not even be convinced of the benefits of it and you would have to try and persuade them that this is something worthwhile.” **[MSD5]**  “It is not just about the end product, it is about everything that goes with it that we need to test and work out too. So, the instructions that we give to people as to how to use it, how we advertise it, who we train in the facility in terms of helping patients to use it, how we promote it to staff so that they know it is available to their patients as well.” **[MSD1]**  “You really need to work with the organization and that is where that whole implementation phase becomes crucial because even if your thing is beautiful, if they do not have the support to make it work, it will fall down.” **[MSD3]** |
| **Guideline 2:**  Planning for and assessing health behavior change | “You have to engage in the behavior change literature […]. A health practitioner probably knows that there is behavior change literature to go to, but someone outside that health domain may not know to go to that literature.” **[MSD8]**  “I do not think it necessitates psychologists or behavioral scientists, but it does necessitate […] looking into those fields. I do not think it is something that you necessarily need to be an expert on as long as you know that that is important, and then you know where to look. And I do think there is a particular stage where it should be involved and that is right at the start.” **[MSD1]**  “You are talking about changing behaviors that are there for a reason. They are not just trivial behaviors, they are deeply embedded and they have really unusual reasonings that […] surprise you. Whereas if you are just designing a booking system or whatever, it is not that emotive.” **[MSD6]**  “One of the main challenges is that the way co-design frameworks [are] very focused on picking a series of methods for a workshop, and then saying, ‘okay participants, I all want you to do this method using these kinds of materials.’ [This] is just completely unfeasible when you have people with only one hand […]. Co-design […] for healthcare […] does have to be approached differently.” **[CME2]** |
| **Guideline 3:**  Identifying and involving co-design facilitators | “In most of these fields usually experts get to make decisions about what should happen and what people should do […], and the very idea that people are the experts in their own lives is a radical idea, especially for a lot of medical professionals, as well as a lot of academic researchers […]. Some of them really struggle with the idea that they may not know what is best for other people. So that idea I think is quite a radical one in co-design. It is not to say there is no place for experts and specialization, but it is to try and even out the playing field a bit more so that people’s lived experience […] are also respected. […] That is a real challenge for people who have been trained to privilege different kinds of expertise and authority.” **[CME6]**  “I think that power balance is particularly interesting in healthcare because it is really hard to say that you do not agree [with] a doctor. […] They are held up in such high esteem as being experts of the subject matter […]. So, to then put-up patients in a room [saying] ‘co-design with your doctors’, it could be really confronting to [say] ‘oh I have a different opinion to you and I do not usually get to express it in my experiences with you, but now can I?’” **[CME2]**  “I think that is probably how we ended up offending people because we did not really know what it is like to give up [smoking], we just sort of went ‘you just give up’. So, we were a little bit disconnected from knowing what it truly means to struggle with [an] addiction that you want to give up and you know is bad for you […]. All these issues are quite emotional and unless you understand how it really feels I think it is important for whoever is running the workshop [to] have a feel for the topic, a knowledge of what it means.” **[MSD6]**  “I think the more practical power distance issues in sessions can be easily navigated if you just have a bit of a soft human touch to ensure that people do not feel like you are the cocky arrogant researcher, expert, designer, or however you are positioning yourself.” **[CME4]**  “There is comfort that comes from people who are like you. [This] is why I saw the two [facilitators] being so successful with the low self-esteem kids because they themselves started the session talking about their problems. The designers running the session were able to talk about their experiences and how they dealt with it and so then they immediately became not the person leading the co-design activity, but a true co-designer.” **[MSD6]**  “There are a few researchers that I do not necessarily put in a room because I know that they will probably rather drag down the vibe than really give the participants the feeling that we are here to […] make stuff happen and that our ideas and our input is valuable […]. A lot has to do with the facilitators in the way they kick off a session, the way they talk to the participants, the way they deal with the participants. That is obviously one important element and that can make a massive difference.” **[CME4]** |
| **Guideline 4:**  Immersion into the mHealth ecosystem | “You do have to be embedded in the space in order to identify it, or you have to be listening to people who are embedded in the space in order to identify it.” **[MSD1]**  “When you work with other people you have to immerse in their situation.” **[CME3]**  “We started with a empathize phase, [which] was around interviewing all different types of stakeholders individually to try and understand what their experiences are, what their frustrations are, what their behaviors and pain points are, what they really struggle with […]. If I had my time again, I would have some kind of hospital administrator, someone that had power within their health service to give us feedback in terms of what would work in planning for the implementation phase.” **[MSD2]**  “I would say involve them right from the start. […] Ascertain to what extent they are going to be able to contribute any of their time […] and ask them what stage they think they want to be involved […] and let them drive that process.” **[MSD5]**  “Ethics was a real challenge. I think we were brought in too late. […] I think that you need to bring the designers in as early as possible. […] What I would have done is get us involved before the ethics application went in. Engage somebody who has got co-design expertise to help shape the ethics, ideally the people that you want to commission to do the work.” **[MSD3]**  “I think it was essential to have [app developers] involved [at the beginning] because it meant that they could identify what was most important because they are the experts in developing this tech. We do not know how to do it. […] We were there as experts in health services and in research, and the consumers and the other stakeholders were there from their expert perspectives, but we needed the app developers and IT and health management as well because we wanted it to be a viable solution. So blue sky thinking is one thing, but we wanted it to happen as well. So, we had to be aware of what limitations were there.” **[CME7]**  “The best way to manage the different stakeholders and the management is at different stages [to] highlight the appropriate stakeholders that are necessary and let them know what their voice is and what their purpose is. Basically, letting stakeholders know when their input is important and needed and what the reason for their input is.” **[CME8]**  “The problem should really be generated in part by the people who are affected when it has something to do with health management. That means that there is a need for more discussion early on. It also means there must be more of an ongoing relationship [with end-users] even if there is not a particular problem yet. Just like maintaining a relationship with the consumers that you work with is important because you do not know what might come up.” **[MSD1]**  “I get really concerned when I see just one or two people with lived experience brought on as kind of the token users to a predominantly professional group and you just think how can those people feel confident and comfortable in that setting, especially in a health context where they are used to being told by the professionals.” **[CME6]**  “You do need to have a kind of hierarchy of who your stakeholders are I suppose. So, the app developers are IT stakeholders, but really our most important stakeholders were the patients and so I would tell someone, remember your hierarchy.” **[MSD1]** |
| **Guideline 5:**  Identifying and involving post-design advocates | “Implementation of mHealth tools is extraordinarily challenging […]. There are a lot of barriers of getting things into practice and getting that buy-in from communities […] can actually aid your implementation because they have already bought in. Because they are invested in it, they are more likely to try and help make it happen.” **[MSD7]**  “If you are integrating into health systems, that has its own challenges, and the other thing is actually getting people to use and promote these tools. So, we talk about champions, you have got to have people behind it that are going to drive it and push it. They are going to refer patients or their communities to it, or they are going to support services to use these tools” **[MSD7]**  “It is not just about the end product, it is about everything that goes with it that we need to test and work out too. So, the instructions that we give to people as to how to use it, how we advertise it, who we train in the facility in terms of helping patients to use it, how we promote it to staff so that they know it is available to their patients as well.” **[MSD1]**  “If they were not taught how to use [the app] properly, if they were not given the right support materials, or if it did not get to the right people because the people who did the roll out of it were not briefed well enough around the sorts of people we want it to go to, even if it was really beautifully designed, then it would have failed. So, I am talking about the wraparound services of the thing. It is not just a thing.” **[MSD3]**  “You will find some end-users in this process who are really interested in what you are doing and how you are doing it and what it could mean for them. Those are the kind of people who might become your post-design advocates who would collect this data for you and at a reasonable price because they have a vested interest in seeing how it worked and helping other people manage their lives for example. So, you could build it into the whole process.” **[CME1]** |
| **Guideline 6:**  Applying health-specific evaluation criteria | “Even though your interruption through technology might end up with things being better, you still have to be very conscious of the fact that there is more at stake if anything goes wrong because I would not want to be involved in a technology that made things more complicated for people who are already in a complicated and stressful situation.” **[MSD1]**  “In healthcare you cannot give patients a medical device or something that might impact on their health without going through the appropriate ethical channels.” **[MSD2]**  “It is then classified as a medical device. So that is a whole different process in terms of implementation. The health service might not be interested in implementing something that is not proven. So, you need a randomized controlled trial of the app first, so that is in the evaluation phase, not the implementation phase, to then prove that it increases patient outcomes and then they might adopt it.” **[MSD2]**  “You definitely need user engagement and prototype testing before [the mHealth system] is deployed. You want to make sure your product is as good as that can be before it can be deployed, particularly for mHealth interventions where it can be accessed readily by a large population. You need a lot of consumer testing or prototype testing before it is deployed.” **[MSD8]**  “You would not naturally do a clinical trial or a randomized control trial in your implementation phase because you first need to be able to test the feasibility. So, you would not do the clinical trial or the randomized control trial until you have got some pilot data back and done it. I would split that up because they are high-level quality study designs. You would only save that for once you have got some pilot data. We would never as a health researcher or a health clinician move straight into a randomized controlled trial without pilot data first […]. In terms of costings, randomized control trials are much more expensive to run and they are the gold star or grade one evidence.” **[MSD8]**  “You have to have a pilot test of it for feasibility of deploying it in the real world because there is going to be quite a difference between what you have generated in the generative phase and what can actually be taken up day-to-day in the real world. […] You need to do it in stages, especially because there is such a massive cost involved in terms of the upkeep of apps and other technologies too. If you can have a prototype, it is not just about testing the prototype, it is also about testing how the prototype works in the real world before you turn it into the end product” **[MSD1]** |
| **Guideline 7:** Collecting and analyzing usage data to understand impact | “I think post-implementation and the collection of evidence of the impact of that change is absolutely essential because you are talking about people changing their behavior for better health outcomes.” **[CME7]**  “[The impact,] you do not necessarily know until something is out in the real world.” **[CME6]**  “In this post-implementation phase, […] you can get post-design interviews and scenarios, but the additional part of that is that you have got all these functionality and metrics that you can get from mHealth that you cannot get anywhere else [such as] Google metrics, Google Analytics, and usage statistics […]. That is a whole avenue of data that you do not have when you do not have mHealth.” **[MSD8]**  “There are ways to get feedback, like usage statistics. Those do not tell you why. Having more qualitative methods to get feedback is really important.” **[CME6]**  “All apps need to be updated, and one of the biggest issues with health apps is they are not.” **[MSD7]**  “You do not just put something out in a digital form and it is done.” **[CME6]** |
