# Supplementary material for: Using Co-design in Mobile Health System Development: A Qualitative Study With Experts in Co-design and Mobile Health System Development
Source: JMIR Mhealth Uhealth. 2021 Nov 10;9(11):e27896. doi: 10.2196/27896 (PMC8663505; doi:10.2196/27896)
Supplement: Multimedia Appendix 5 [file mhealth_v9i11e27896_app5.docx]

## Multimedia Appendix 5: Checklist for System Designers

This table provides a checklist for the adoption of co-design in a mHealth systems project along with notes on the involved activities and stakeholders.

| **Table 1.** Checklist for System Designers | |
| --- | --- |
| □ | **Guideline 1:** *Carefully consider the unique circumstances of the targeted disease management or health promotion context with respect to its evaluation and integration requirements, stakeholder involvement, and end-user vulnerabilities relating to highly personal aspects of a person’s health.* |
|  | **Notes:** |
| □ | **Guideline 2:** *As early as possible in the co-design process, consult the behavior change literature and/or involve experts in behavior change relevant to the problem context to effectively identify the targeted change in behavior and adequately plan the type and stakeholder involvement of co-design activities.* |
|  | **Notes:** |
| □ | **Guideline 3:** *Select and engage co-design facilitators that have an authentic understanding of the intimate problem context (e.g., first-hand experience, immersing in problem context, literature consultation), and operate in an empathetic way to mitigate potential barriers associated with the power distance between mHealth stakeholders. Immerse* |
|  | **Notes:** |
| □ | **Guideline 4:** *yourself in the underlying complex health context to identify and understand stakeholders early, include them in defining their involvement in the co-design process along existing health process requirements, recognize the diversity and power distances among stakeholders, and prioritize the needs of the end-user.* |
|  | **Notes:** |
| □ | **Guideline 5:** *Throughout every phase of co-design, identify potential post-design advocates from different stakeholder categories who can aid in implementing the mHealth system (e.g., training staff in the use of the system) and championing its usage in the post-design phase (e.g., providing feedback on system usage in practice).* |
|  | **Notes:** |
| □ | **Guideline 6:** *In the evaluative phase, ensure that the mHealth system goes through feasibility testing in the real world (pilot testing and randomized control trials) to adequately address ethical considerations in the health context, determine potential risks to the end-users caused by the artifact, and clarify whether it accomplishes its intended goals before implementation.* |
|  | **Notes:** |
| □ | **Guideline 7:** *In the post-design phase, collect usage data to observe the mHealth system’s impact after it has been implemented and apply contextual co-design methods to understand this impact.* |
|  | **Notes:** |
